# *P2rx1* deficiency alleviates acetaminophen-induced acute liver failure by regulating the STING signaling pathway

**DOI:** 10.1007/s10565-023-09800-1

**Published:** 2023-04-13

**Authors:** Yeping Yu, Ling Chang, Qingluan Hu, Jianjun Zhu, Jianjun Zhang, Qiang Xia, Jie Zhao

**Affiliations:** 1 Department of Liver Surgery, Renji Hospital, School of Medicine, Shanghai Jiao Tong University, Shanghai, 200127 China; 2 Department of Gastroenterology, Seventh People’s Hospital of Shanghai University of Traditional Chinese Medicine, Shanghai, China

**Keywords:** P2RX1, APAP, Mitochondria dysfunction, STING, Acute liver failure

## Abstract

**Aims:**

Purinergic signaling-mediated mitochondria dysfunction and innate immune-mediated inflammation act as triggers during acetaminophen (APAP)-induced liver injury (AILI). However, the underlying mechanisms by which purinoceptor regulates mitochondria function and inflammation response in the progression of AILI remains unclear.

**Methods:**

First, the hepatic level of purinergic receptor P2X 1 (P2RX1) was identified in the DILI patients and APAP-induced WT mice. *P2rx1* knockout (KO) mice (*P2rx1*^−/−^) with 300 mg/kg APAP challenge were used for the analysis of the potential role of P2RX1 in the progression of AILI. Administration of DMX, the activator of stimulator of interferon genes (STING), was performed to investigate the effects of the STING-related pathway on APAP-treated *P2rx1*^−/−^ mice.

**Results:**

The elevated hepatic P2RX1 levels were found in DILI patients and the AILI mice. *P2rx1* depletion offered protection against the initial stages of AILI, mainly by inhibiting cell death and promoting inflammation resolution, which was associated with alleviating mitochondria dysfunction. Mechanistically, *P2rx1* depletion could inhibit STING-TANK-binding kinase 1 (TBK1)-P65 signaling pathways in vivo. We then showed that DMX-mediated STING activation could greatly aggravate the liver injury of *P2rx1*^−/−^ mice treated with APAP.

**Conclusion:**

Our data confirmed that P2RX1 was inducted during AILI, identified P2RX1 as a novel regulator in mitochondria dysfunction and STING pathways, and suggested a promising therapeutic approach for AILI involving the blockade of P2RX1.

**Graphical abstract:**

1. It first demonstrated the protective effects of *P2rx1* deficiency on acetaminophen-induced liver injury (AILI).

2. *P2rx1* knockout alleviates mitochondria function and promotes inflammation resolution after APAP treatment.

3. It first reported the regulation of P2RX1 on the STING signaling pathway in the progress of AILI.

4. P2RX1 blockade is a promising therapeutic strategy for AILI.

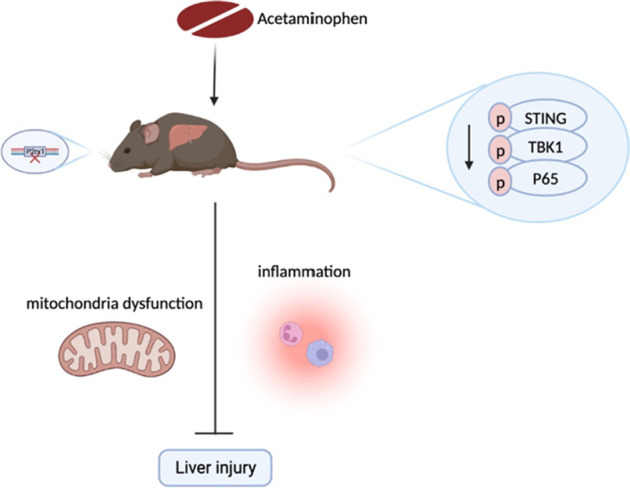

## Introduction

In the US and other western countries, acetaminophen ﻿(APAP) overdose is the most common cause of drug-induced liver injury (DILI), the leading reason for acute liver failure (ALF) (Jaeschke et al. 2021). Due to the scarcity of acceptable donor tissue and the ensuing lifelong immunosuppression, liver transplantation may not be the most applicable treatment option for DILI patients who eventually develop ALF. It can be seen that the creation of new strategies or objectives that reduce APAP-induced liver damage is urgently required.

Cytochrome P450 2E1 (CYP2E1) catalyzes the conversion of APAP into N-acetyl-p-benzoquinone imine (NAPQI), a reactive metabolite that quickly conjugates with glutathione (GSH) (Yuan and Kaplowitz 2013). Once the quantities of GSH are restricted, excess NAPQI combines with hepatocellular components to generate NAPQI-protein adducts (APAP-protein adducts), which in turn causes mitochondrial dysfunction. Excessive reactive oxidative stress, symbolized by oxygen species (ROS) production, is recognized as an important pathological process of the APAP-induced liver injury (AILI) (Burke 2017). In response to particular signals such as ROS, the outer membrane of mitochondria becomes sufficiently permeable to release apoptosis-inducing proteins and accelerate cell death (Gustafsson and Gottlieb 2003). On the other hand, the proton gradient collapses and ATP production is disrupted as a result of the mitochondrial permeability transition pore (mPTP) opening (Gustafsson and Gottlieb 2008; Chiong et al. 2011). Hence, specific steps of cell death require ATP and mitochondrial dysfunction under hepatic damage, suggesting the essential role of mitochondrial function in the AILI (Eguchi et al. 1997).

Purines and purine nucleotides are universal intracellular energy currencies for biological reactions, whose roles in liver function and responses to inflammation have also been highlighted (Vaughn et al. 2012, 2014; Eltzschig et al. 2012; Vuerich et al. 2019). Two kinds of purinoceptors known as purinergic 1 (P1) receptors, by adenosine, and purinergic 2 (P2) receptors are essential for controlling a variety of cellular processes, such as growth, death, and metabolism. Purinergic 1 (P1) receptors, ﻿triggered by adenosine, and purinergic 2 (P2) receptors have been identified as ﻿two families of purinoceptors ﻿that are essential for controlling a variety of cellular processes, such as growth, death, and metabolism (Allard et al. 2017; Virgilio et al. 2018). The P2 receptor family consists of seven ATP-gated channel P2X members ﻿(P2RX1–7) and eight subtypes of Ca^2+/^phosphatidylinositol linked P2Y receptors (P2RY) triggered by ATP and nucleotides. P2RX1 is expressed in a wide variety of cells and has impacts on inflammation, wound healing, scar tissue formation, and malignancy (Virgilio and Adinolfi 2017). Genetic knockout of *P2rx1* has been reported to be beneficial to inflammatory bowel disease by aryl hydrocarbon receptor (AhR)/IL-22 axis involved in microbiota metabolites (Wang et al. 2021). Moreover, Wang et al. found the formation of neutrophil extracellular traps (NETs), promoting the disturbance of mitochondrial dynamics in IRI, was critically dependent on the P2RX1-involved metabolic contact between platelets and neutrophils (Zhuang et al. 2021). Although the cumulative studies confirmed that P2RX1 played an important role in mitochondria dynamics, the detailed effects of P2RX1 on AILI remain unclear.

Accumulated evidence has suggested that pathogenic DNA, especially mitochondria DNA (mtDNA), could be sensed by the cyclic GMP-AMP synthase (cGAS) (Yu et al. 2022). cGAS then activates the stimulator of interferon genes (STING) signaling pathway, which was confirmed to be related to the liver against APAP-induced inflammatory responses and apoptosis (Chen et al. 2022). STING may act as a switch between cytosolic DNA sensor and innate immune response according to present data. STING pathway-related inflammation in macrophages leads to the progression of nonalcoholic fatty liver disease (Luo et al. 2018). Multiple strategies for modulating STING, including genetic control of the STING pathway, alternative splicing, and post-translational modification, have been investigated (Zhang et al. 2022). However, it has not been reported whether P2RX1 affects STING-related pathways, although many regulation mechanisms for STING pathways have been identified.

Here, we examined the effect and underlying mechanism of *P2rx1* deficiency regulating mitochondria function and STING signaling-mediated inflammation in a mouse model of AILI. Together, our findings offer new insights about which purinergic receptor, especially P2RX1, could be a promising strategy to balance inflammation and mitochondria function during the progression of AILI by regulating the STING signaling pathway.

## Methods

### Human liver species

Three DILI patients undergoing liver biopsy and three healthy donors (HC) were recruited at the Department of Liver Surgery, Renji Hospital, School of Medicine, Shanghai Jiao Tong University. Liver samples were collected and used with the approval of the Ethics Committee of Renji Hospital.

### Mice

*P2rx1* knockout (KO) mice (*P2rx1*^−/−^) constructed with the guidance of the CRISPR/Cas9 system were obtained from ﻿GemPharmatech Co. ﻿Ltd. (Jiangsu, China). The strains of *P2rx1*^−/−^ and wild-type (WT) mice used in the experiments were of the C57BL/6 J variety and were kept in specific pathogen-free environments with standard light cycles, temperature, and humidity controls. Male 6–8-week-old *P2rx1*^−/−^ and WT mice were fasted for 15–17 h before receiving an i.p. injection of either a single model dose of freshly made APAP (300 mg/kg, Sigma-Aldrich, St. Louis, MO), considered as the﻿ experimental group (n = 4–6) or PBS (control group, n = 4–6). At the 6 h mark following the APAP challenge, blood samples, and liver tissues were harvested immediately. A single lethal dose of APAP (500 mg/kg) was injected into these two genotypes (n = 12) for the analysis of survival rate. Mice were treated intraperitoneally with 10 mg/kg DMXAA (﻿abbreviated as DMX, an agonist of STING, Selleck) ﻿or DMSO 2 h before PBS or 300 mg/kg APAP injection (n = 4–6) to explore the potential mechanism of *P2rx1* knockout. The experiments involving animals were conducted with the approval of Renji Hospital's Institutional Animal Care and Use Committees.

### Serum indicators analysis

Serum was extracted from blood samples after centrifuging at 3,000 rpm for 10 min. Serum alanine transaminase (ALT) and aspartate transaminase (AST) were quantified by TestKit (Nanjing Jiancheng Bioengineering Institute, Nanjing, China). Enzyme-linked immunosorbent assay (ELISA) kits (Multi-Science, Hangzhou, China) were used to quantify serum tumor necrosis factor (TNF)-α, interleukin (IL)-6, and macrophage chemoattractant protein (MCP)-1 indicators according to the manufacturer’s protocols.

### Liver hematoxylin and eosin (H&E), immunohistochemical (IHC), immunofluorescence (IF), and TUNEL staining

Over 24 h of fixation in 4% paraformaldehyde, paraffin embedding, and 5 m thick slices of liver tissues were performed as standard procedures. The liver sections were stained with H&E to analyze the histopathological changes. As for the materials, incubations were carried out with these primary antibodies specific for P2RX1 (1:200; ﻿Alomone), CD11b (1:200; Abcam), MPO (1:300; Abcam), and cleaved-caspase-3 (1:50; Abcam). DAPI-containing media (Vector Labs) and DAB substrate kit (Abcam) were made use for IF and IHC staining, representatively.

### Quantitative polymerase chain reaction (qPCR)

qPCR was performed as previously described (Li et al. 2020a). 2(^−ΔΔCt^) technique was used to calculate relative fold changes in target gene expression when *Gapdh* acted as a standard. ﻿The following primers were provided: ﻿*P2rx1*-F: GGATGGTGCTGGTACGAAACA, *P2rx1*-R: CACTGACACACTGCTGATAAGG; *Tnf-α*-F: CCCTCACACTCAGATCATCTTCT; *Tnf-α*-R: GCTACGACGTGGGCTACAG; *Il-6*-F: TAGTCCTTCCTACCCCAATTTCC; *Il-6*-R: TTGGTCCTTAGCCACTCCTTC; *Mcp-1*-F: CAGATGTGGTGGGTTTCTCATAGCC; *Mcp-1*-R: GCTCCAAGGGTGACAGTGATTTCTC; *Gapdh*-F: AGGTCGGTGTGAACGGATTTG; *Gapdh*-R: TGTAGACCATGTAGTTGAGGTCA.

### RNA sequencing (RNA-seq) analysis

Total RNA from APAP injured WT and *P2rx1*^−/−^ liver tissues was extracted using TRIzol (Invitrogen, Carlsbad, CA, USA) and RNeasy Kit (Qiagen, Hilden, Germany), which was subsequently sent to Shanghai Neo-Biotechnology Co., Ltd for clustering and sequencing. Gene expression was calculated according to the FPKM value. The contribution of signaling pathways to the phenotype was evaluated using KEGG analysis and gene set enrichment analysis (GSEA).

### mtDNA measurement

qPCR was used to quantify mtDNA, as described previously (Wen et al. 2019). Total DNA was isolated from plasma samples using QIAamp Blood and Mini Kit (Qiagen, CA, USA). The qPCR study was carried out using the following primer sets, designed for the mouse cytochrome c oxidase subunit III, and the same amount of DNA diluted in each reaction system: F: ACCAAGGCCACCACACTCCT; R: ACGCTCAGAAGAATCCTGCAAAGAA. mtDNA was extracted from mouse liver as standard, and its purity was confirmed by qPCR with primers specific to mitochondrial genes.

### Liver malonaldehyde (MDA) measurement and Caspase-3 Activity Assay

Hepatic MDA concentrations and Caspase-3 activity of fresh liver tissues were measured using a commercial kit (Beyotime, Shanghai, China) and a Total MDA Quantification kit (Nanjing Jiancheng Bioengineering Institute, Nanjing, China) respectively.

### Western blot

As previously reported, liver tissues or cultured cells were processed for western blot analysis (Li et al. 2020b). Following primary antibodies specific for P2RX1 (1:200; Alomone), BCL-2, BCL-XL, STING, p-STING, TANK-binding kinase 1 (TBK1), p-TBK1, P65, and p-P65 (1:1000; Cell Signaling Technology) have been utilized. The loading control was β-ACTIN (1:10,000; Sigma-Aldrich).

### Primary hepatocyte isolation and treatment

Collagenase perfusion was used to separate primary hepatocytes of WT or *P2rx1*^−/−^ mice (Klaunig et al. 1981). In a fresh, serum-free, antibiotic-free DMEM culture media, hepatocytes were starved for 12 h before 5 mM APAP or PBS treatment in vitro for 6 h. Hepatocyte death was assessed by TUNEL staining. MitoSOX Red dye (Multi-Science, Hangzhou, China) and JC-1 fluorescent dye (Multi-Science, Hangzhou, China) were characterized to assess the extent of mitochondria ROS and mitochondrial membrane potential. Isolated hepatocytes were treated with 75 μg/mL DMX ﻿or DMSO 2 h before PBS or 5 mM APAP to explore the effects of STING agonist on mitochondria.

### Statistical analysis

Data were shown as the mean ± SEM. Student’s t-tests and ﻿one-way analysis of variance (ANOVA) were used to analyze the data. The log-rank (Mantel-Cox) test was employed to compare the survival. Statistical analysis was conducted on GraphPad Prism 8 (GraphPad Software, San Diego, CA, USA). ﻿p values below 0.05 were deemed to be significant.

## Results

### Knockout of *P2rx1* alleviates liver injury in the AILI mouse model

**Fig. 1:**
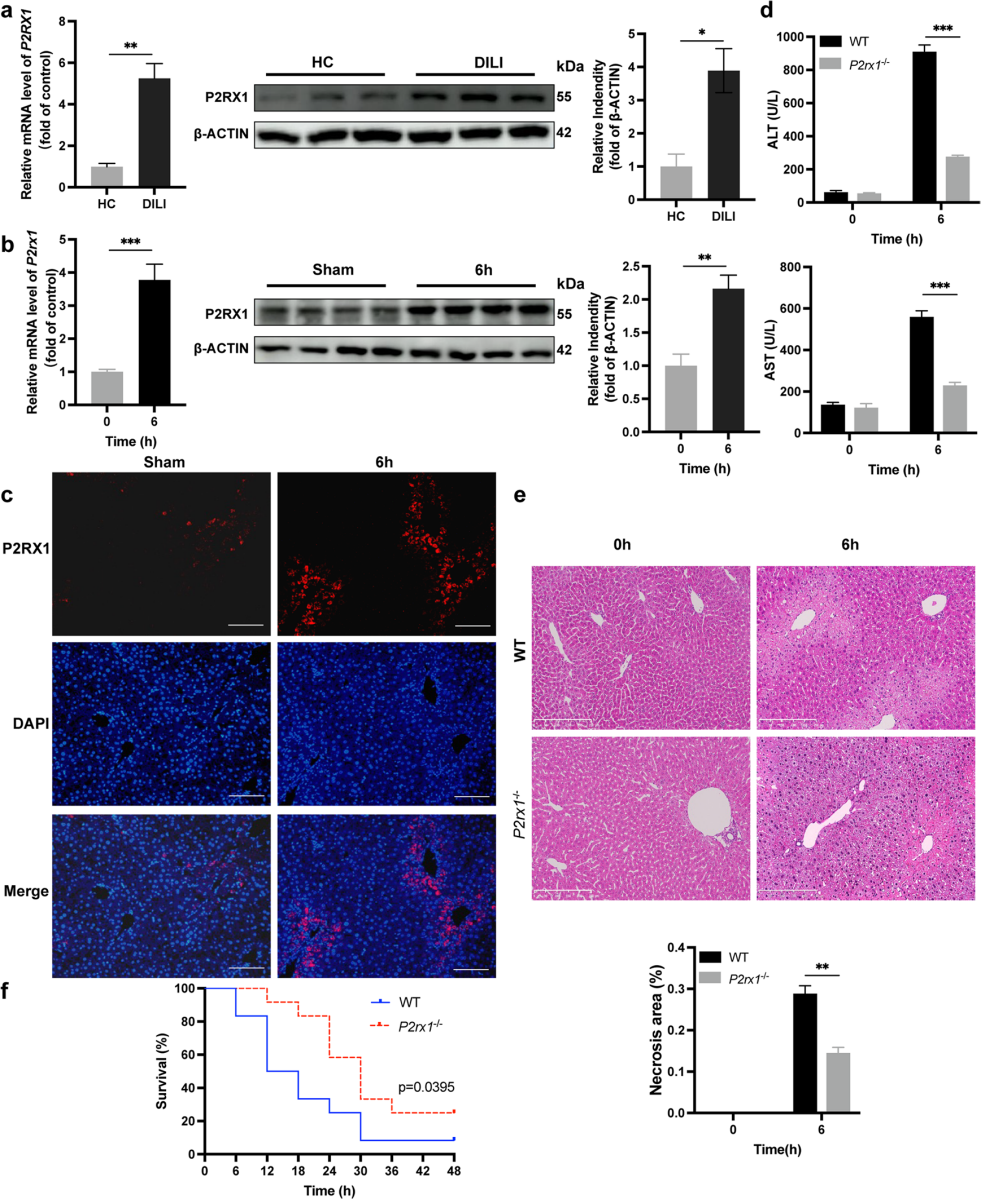
Knockout of *P2rx1* alleviates liver injury in the AILI mouse model. Dynamic hepatic relative *P2rx1* mRNA expression and P2RX1 expression in (a) DILI patients and (b) APAP-treated WT mice (n = 4–6 per group); (c) Representative IF staining images of P2RX1 in WT mice with or without AILI (origin magnification × 100, scale bar = 100 μm); (d) Serum ALT and AST levels in WT and *P2rx1*^−/−^ mice after a single dose of PBS or APAP (300 mg/kg, n = 4–6 per group); (e) Representative images of H&E staining in liver sections of WT and *P2rx1*^−/−^ mice with or without APAP challenge (origin magnification × 100). Quantification of necrotic areas in liver sections by H&E staining (n = 4–6 per group); (f) Survival curve of WT and *P2rx1*^−/−^ mice in response to a single lethal dose of APAP (500 mg/kg, n = 12 per group). Data are shown as the means ± SEM, *p < 0.05, **p < 0.01, ***p < 0.001

qPCR and western blot analysis of liver samples from DILI patients revealed an upward increase in P2RX1 expression, compared with HC (Fig. 1a). The hepatic P2RX1 levels rose dramatically 6 h after a single dose of APAP, revealed by both qPCR and western blot (Fig. 1b). Liver sections stained with IF for P2RX1 showed that P2RX1 was significantly induced by APAP (Fig. 1c). These findings imply the elevated level of P2RX1 in the DILI patients and AILI model.

To investigate the possible relevance of P2RX1 in AILI, a single dose of APAP (300 mg/kg) was used to induce liver injury paradigm in both WT and *P2rx1*^−/−^ mice. Compared to WT mice, serum ALT/AST elevation (Fig. 1d) and hepatic necrosis regions (Fig. 1e) after APAP exposure were significantly reduced in *P2rx1*^−/−^ mice, indicating diminished liver damage. To screen if *P2rx1* depletion protected mice from mortality*,* mice were given a larger single dose of APAP (i.p., 500 mg/kg). After 48 h, 91.67% of WT mice (n = 12) succumbed to the lethal dose of APAP, while the survival rate of *P2rx1*^−/−^ mice (n = 12) was 25% (Fig. 1f). *P2rx1*^−/−^ mice subjected to an overdose of APAP gained remarkable improvement in survival. The evidence presented here strengthens the hypothesis that *P2rx1* depletion ameliorates AILI.

### *P2rx1* depletion eliminates APAP-induced cell death

**Fig. 2:**
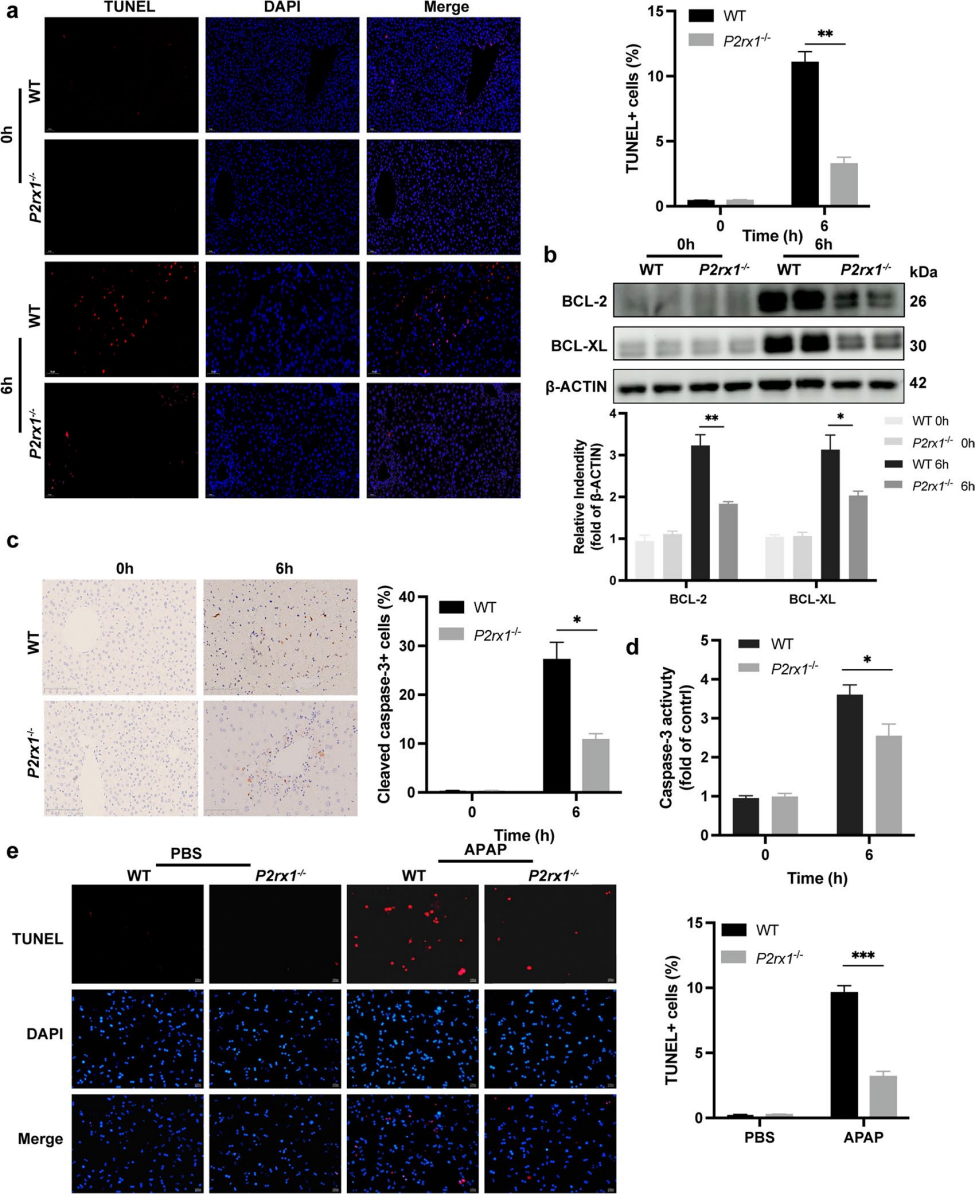
*P2rx1* depletion eliminates APAP-induced cell death. (a) Representative images of TUNEL staining (original magnification × 200) and the statistical quantification of TUNEL-positive cells in liver sections of WT and *P2rx1*^−/−^ mice with or without APAP treatment (n = 4–6 per group); (b) Western blot and quantification analysis for the expression of hepatic BCL-2 and BCL-XL in WT and *P2rx1*^−/−^ mice with or without APAP treatment (n = 3–4); (c) ﻿Representative IHC staining (original magnification × 200) and the statistical quantification of hepatic cleaved-caspase-3-positive cells in liver sections of WT and *P2rx1*^−/−^ mice with or without APAP treatment (n = 4–6 per group); (d) Caspase-3 activity in the livers with or without APAP treatment (n = 4–6 per group); (e) Representative images of TUNEL staining (original magnification × 200, scale bar = 353 μm) and the quantification of TUNEL-positive primary hepatocytes with or without 5 mM APAP treatment in vitro (n = 3–6 per group). Data are shown as the means ± SEM, *p < 0.05, **p < 0.01, ***p < 0.001

Accumulating evidence indicates that hepatocyte death plays an initial role in the APAP-induced hepatotoxicity (Chen et al. 2019). ﻿We examined if *P2rx1* deficiency diminishes cell death response to APAP treatment. At 6 h after APAP administration, TUNEL staining was markedly reduced in the liver tissues of APAP-treated ﻿*P2rx1*^−/−^ mice (Fig. 2a). Markers of APAP-induced cell death, BCL-2, as well as BCL-XL, were abolished in *P2rx1*^−/−^ mice (Fig. 2b). Cleaved-caspase-3 expression was considerably lower in *P2rx1*^−/−^ mice following APAP exposure compared to WT mice as illustrated by IHC analysis (Fig. 2c). *P2rx1*^−/−^ mice showed a significant reduction in ﻿Caspase-3 activity (Fig. 2d). At 6 h post-APAP (5 mM) stimulation, primary hepatocytes from both genotypes were stained with TUNEL to determine the degree of cell death. In response to the APAP challenge, the number of TUNEL-positive hepatocytes was significantly lower in *P2rx1*^−/−^ mice (Fig. 2e). These data indicate that hepatocytes, in particular, are protected from APAP-induced cell death when *P2rx1* is absent.

### *P2rx1* depletion enhances inflammation resolution in response to APAP treatment

**Fig. 3:**
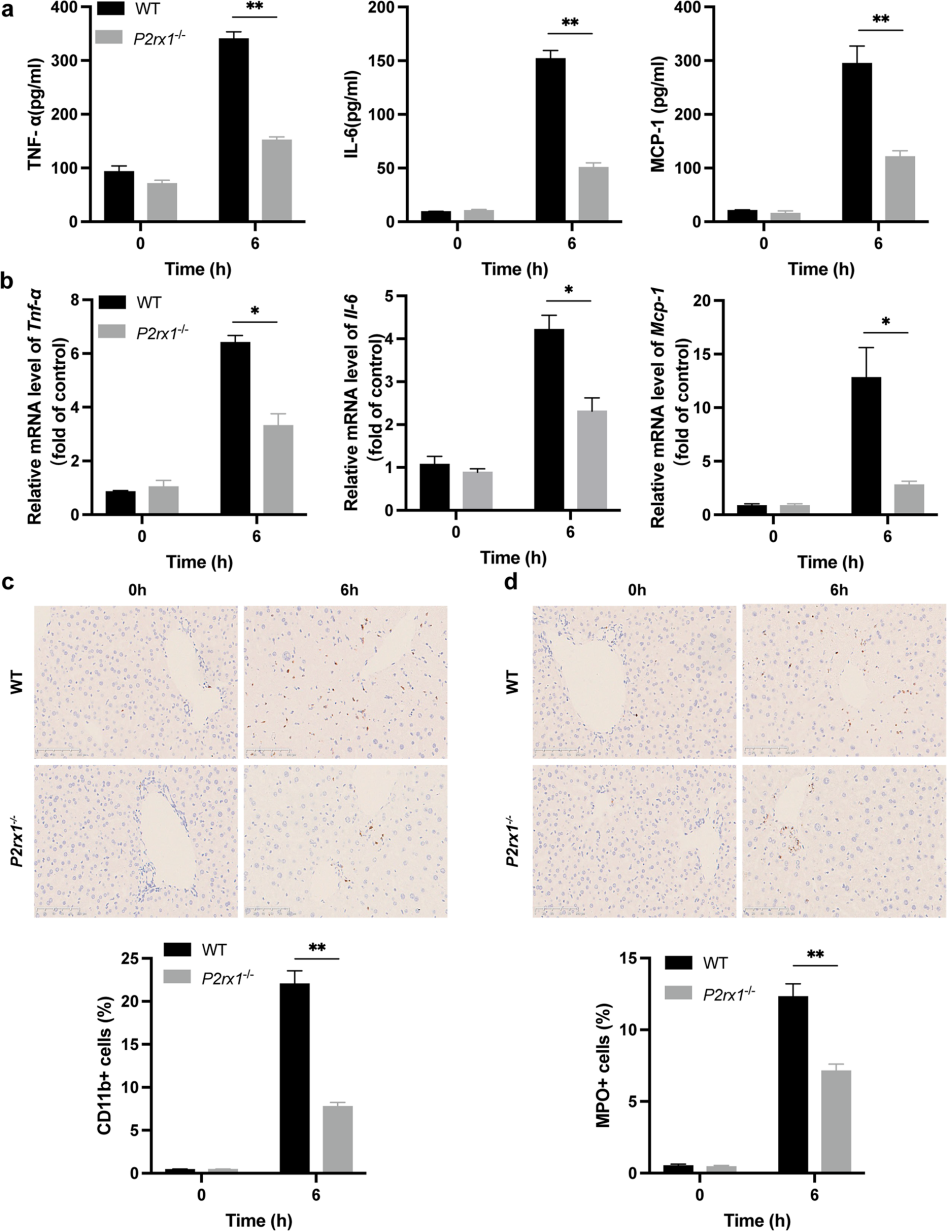
*P2rx1* depletion enhances inflammation resolution in response to APAP treatment. (a) Serum TNF-α, IL-6, and MCP-1 levels of both genotypes after PBS or APAP treatment (n = 4–6 per group); (b) Relative ﻿hepatic *Tnf-α*, *Il-6*, and *Mcp-1* mRNA in both mice after PBS or APAP treatment (n = 4–6 per group); Representative IHC images and the quantification of (c) ﻿CD11b-positive cells and (d) MPO-positive cells in liver sections after PBS or APAP treatment (original magnification × 200, n = 4–6 per group). Data are shown as the means ± SEM, *p < 0.05, **p < 0.01

We probed if the superior liver restoration could be ascribed to the resolution of inflammation in the presence of *P2rx1* blockade, given that disrupted hepatocytes may initiate an immune response, the detrimental signature of AILI (Starkey Lewis et al. 2020). ﻿In *P2rx1*^−/−^ mice, serum levels for TNF-, IL-6, and MCP-1 appeared substantially lower after APAP dosing, as detected by ELISA (Fig. 3a). Challenge for *P2rx1*^−/−^ mice with APAP decreased the hepatic *Tnf-α*, *Il-6*, and *Mcp-1* mRNA levels compared to controls (Fig. 3b). The fewer infiltrating macrophages and neutrophils in the liver of *P2rx1*^−/−^ mice were verified by the IHC for CD11b and MPO (Fig. 3c, 3d). These data indicate that *﻿P2rx1* deficiency might reduce immune cell infiltration and inflammation in response to APAP treatment.

### *P2rx1* depletion improves mitochondria dysfunction and STING signaling-mediated inflammation

**Fig. 4:**
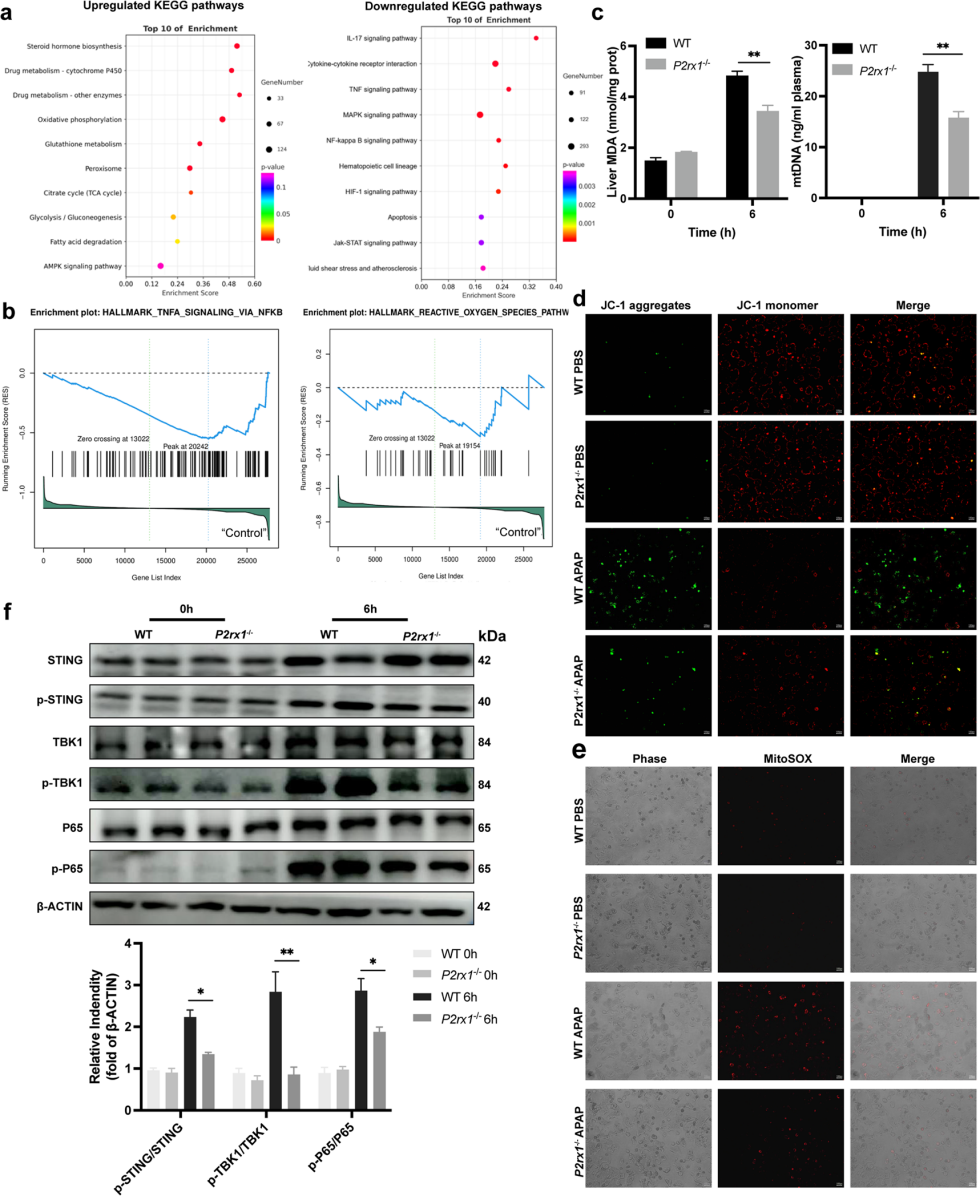
*P2rx1* depletion improves mitochondria dysfunction and STING signaling-mediated inflammation. (a) RNA-seq analysis of livers from mice 6 h after APAP treatment. Bubble chart showing the top 10 of upregulated and downregulated KEGG enrichment of the significant genes; (b) Enrichment plots from the GSEA analysis; (c) Hepatic MDA and plasma mtDNA levels in both genotypes after PBS or APAP treatment (n = 4–6 per group); (d) JC-1 analysis for mitochondrial membrane potentials in primary hepatocytes after PBS or 5 mM APAP treatment (origin magnification × 100, scale bar = 353 μm); (e) Representative images of MitoSOX Red probe in primary hepatocytes after PBS or 5 mM APAP treatment (origin magnification × 100, scale bar = 353 μm); (f) Western blot analysis for expression of the STING-TBK1-P65 signaling pathway in liver extracts of mice after PBS or APAP treatment. Data are shown as the means ± SEM, *p < 0.05, **p < 0.01

RNA-seq analysis was undertaken for both WT and *P2rx1*^−/−^ mice that had been injected with APAP to examine the global transcriptome variations that occur in the presence of AILI. The inflammation resolution and reduced damage-related signaling were upregulated in APAP-induced *P2rx1*^−/−^ mice, as evidenced by a decrease in the expression of genes involved in the NF-κB-mediated pathway, TNF-mediated pathway, and apoptosis during KEGG analysis (Fig. 4a). Notably, *P2rx1*^−/−^ mice experienced a considerable enrichment in pathways involved in mitochondrial function, such as oxidative phosphorylation and the citrate cycle. ﻿Additionally, GSEA analysis revealed that TNF-α signaling via the NF-κB pathway and reactive oxygen species (ROS) pathway showed significant enrichment in gene sets of *P2rx1*^−/−^ mice (Fig. 4b). The remarkable amelioration of mitochondrial dysfunction could be seen by a decrease in hepatic MDA and plasma mtDNA when *P2rx1 was inhibited* (Fig. 4c). To further validate that *P2rx1* depletion resists to mitochondrial dysfunction, JC-1 fluorescent dye and MitoSOX Red dye of isolated primary hepatocytes being exposed to either 5 mM APAP or PBS in vitro were evaluated. Hepatocytes with *P2rx1* depletion showed less mitochondrial depolarization, according to a smaller green/red fluorescence ratio (Fig. 4d). Moreover, ROS accumulated less in *P2rx1*-deficient cells than in their WT counterparts (Fig. 4e).﻿ We then examined the expression of STING-TBK1-P65 pathway in the liver tissues from WT and *P2rx1*^−/−^ mice by western blot analysis (Fig. 4f). ﻿The phosphorylation states of STING, TBK1, and P65 were significantly downregulated in *P2rx1*^−/−^ mice challenged with APAP, indicating the inactive STING signaling pathway in *P2rx1*^−/−^ mice. These findings ﻿suggest that P2RX1 is a novel mitochondria dysfunction regulator and regulates the STING signaling pathway in AILI mice.

### STING activator aggravates liver injury of *P2rx1*^−/−^ mice after APAP treatment

**Fig. 5:**
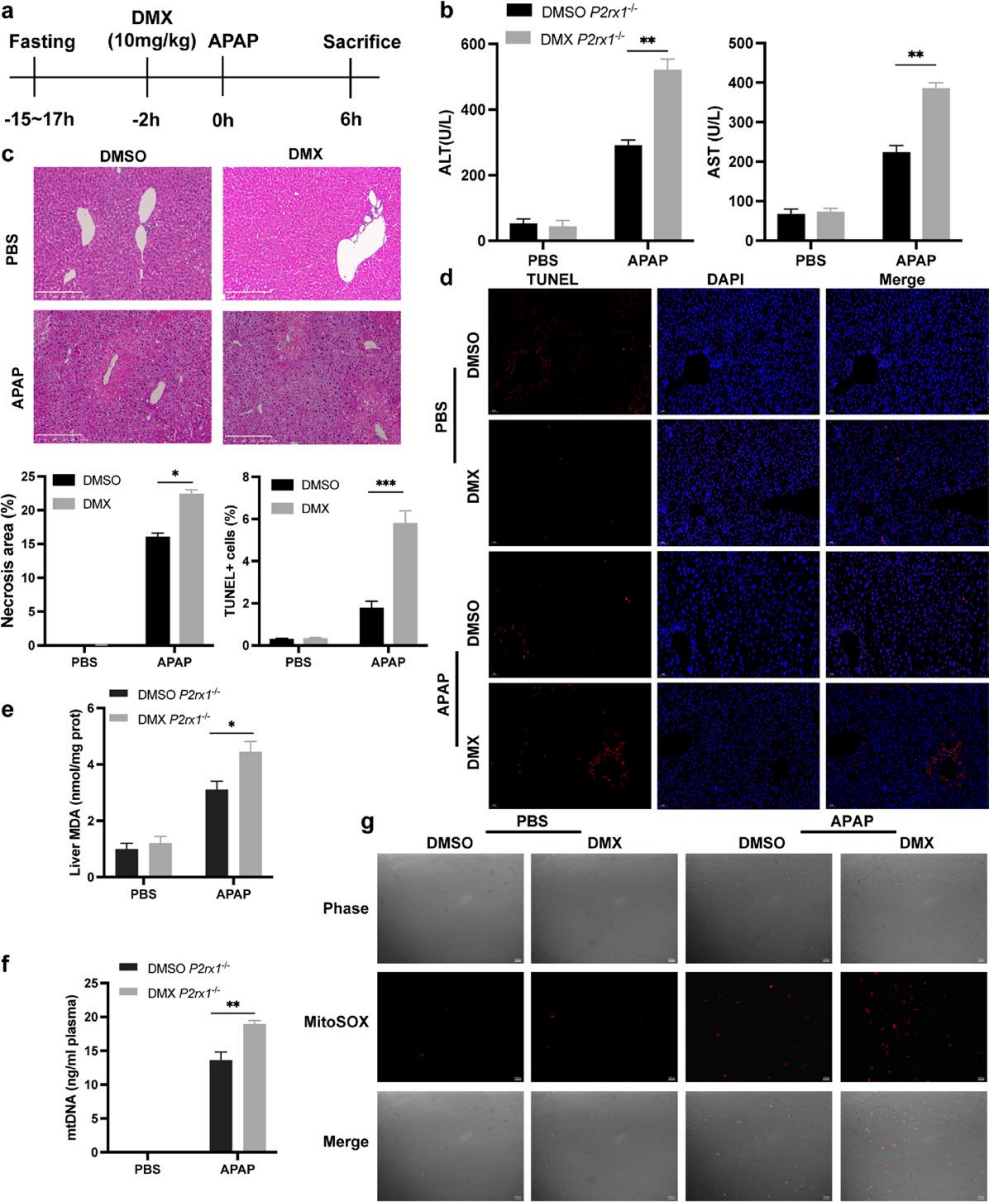
STING activator aggravates liver injury of *P2rx1*^−/−^ mice after APAP treatment. (a) A schematic of STING activation by DMX (10 mg/kg) in the APAP overdose model. (b) Serum levels of ALT and AST in *P2rx1*^−/−^ mice with or without DMX pretreatment (10 mg/kg, n = 4–6 per group); (c) Representative images of H&E staining (original magnification × 100) and quantification of hepatic necrosis area in *P2rx1*^−/−^ mice with or without DMX pretreatment (n = 4–6 per group); (d) Representative images and the quantification of TUNEL-positive cells in liver sections of *P2rx1*^−/−^ mice with or without DMX pretreatment (original magnification × 200, n = 4–6 per group); (e) Hepatic MDA and (f) plasma mtDNA levels of *P2rx1*^−/−^ mice with or without DMX pretreatment; (g) Representative images of MitoSOX Red probe in primary *P2rx1*^−/−^ hepatocytes pretreated with DMSO or DMX. Data are shown as the means ± SEM, *p < 0.05, **p < 0.01, ***p < 0.001

Given that *P2rx1* deficiency reduces mitochondria dysfunction and STING signaling pathway activation, we then confirm the potential mechanism of *P2rx1* depletion in *P2rx1*^−/−^ mice treated with DMSO or DMX (i.p.,10 mg/kg), an activator of STING, 2 h before PBS or 300 mg/kg APAP (Fig. 5a). Compared to DMSO-treated *P2rx1*^−/−^ mice, liver enzymes, necrosis areas, and the percentage of TUNEL-positive cells were all dramatically raised in liver tissues of *P2rx1*^−/−^ mice after DMX administration (Fig. 5b-5d). In addition, increased levels of hepatic MDA, plasma mtDNA, and mitochondrial ROS were observed in DMX-treated *P2rx1*^−/−^ mice, demonstrating the exacerbated APAP-induced mitochondrial dysfunction (Fig. 5e-5g). These results demonstrate that the STING activator may offset the protective effects of *P2rx1* deficiency in AILI mice.

### ﻿STING activator promotes inflammatory response induced by APAP treatment in *P2rx1*^−/−^ mice

**Fig. 6:**
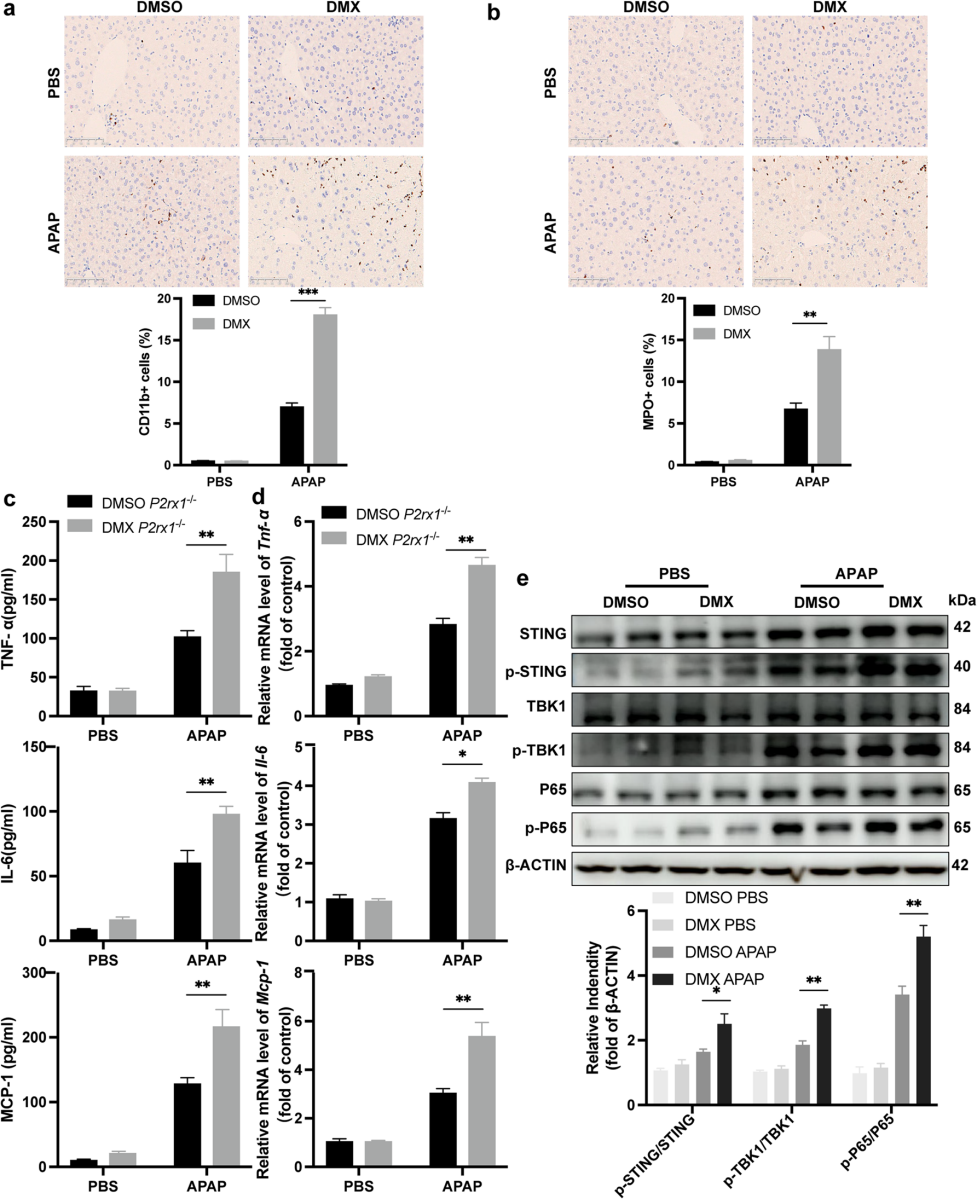
STING activator promotes inflammatory response induced by APAP treatment in *P2rx1*^−/−^ mice. Representative IHC images and qualification of (a) CD11b-positive cells and (b) MPO-positive cells in the liver tissues of *P2rx1*^−/−^ mice with or without DMX pretreatment (origin magnification × 200, n = 4–6 per group); (c) Serum TNF-α, IL-6, and MCP-1 levels of *P2rx1*^−/−^ mice with or without DMX pretreatment (n = 4–6 per group); (d) Relative hepatic *Tnf-α*, *Il-6*, and *Mcp-1* mRNA levels of *P2rx1*^−/−^ mice 6 h with or without DMX pretreatment (n = 4–6 per group); (e) Western blot for the expression of the STING-TBK1-P65 signaling pathway in liver tissues of *P2rx1*^−/−^ mice with or without DMX pretreatment. Data are shown as the means ± SEM, **p < 0.01, ***p < 0.001

Having detected that DMX disrupted liver recovery of *P2rx1*^−/−^ mice after APAP treatment, we evaluated the role of the STING activator on inflammation response in AILI. ﻿As shown in Fig. 6a, 6b, DMX-treated *P2rx1*^−/−^ mice have a massive rise in CD11b-, and MPO-positive cells from liver tissues. Consistent with that, serum levels of TNF-α, IL-6, and MCP-1 were likewise boosted in *P2rx1*^−/−^ mice treated with DMX (Fig. 6c). Synthesis of mRNA for *Tnf-α*, *Il-6*, and *Mcp-1* witnessed a tremendous increase in liver tissues of DMX-treated *P2rx1*^−/−^ mice than those of in DMSO-treated mice (Fig. 6d). Moreover, we confirmed that the STING-TBK1-P65 signaling pathway was activated in the presence of DMX in *P2rx1*^−/−^ mice induced by APAP (Fig. 6e). Mechanically, these results, together with those presented in Fig. 5, suggest that STING-mediated signaling pathway plays a detrimental role in APAP-treated *P2rx1*^−/−^ mice.

## Discussion

Patients with DILI, and especially those with AILI, have a significant hurdle in obtaining a donor liver and surviving the transplant procedure, requiring a novel method to slow or stop the course of liver failure. Here, our research confirmed both individuals with DILI and AILI mouse model showed evidence of P2RX1 induction. Functionally, we found that *P2rx1* deficiency played a therapeutic role in AILI at the early stage by reducing cell death and improving inflammation resolution, which is mainly mediated by innate immune regulation. Mechanistically, *P2rx1* deficiency could ameliorate mitochondria dysfunction, inhibiting the STING-TBK1-P65 signaling pathway, which could be offset by the STING activator. Consequently, we concluded that P2RX1 suppression poses therapeutic potential in AILI caused by APAP overdose.

Hepatic metabolic activities are regulated in normal physiology by the purinergic receptor family of G protein-coupled receptors (GPCRs), which are highly expressed in liver resident cells. Purinergic receptors, among the family of G protein-coupled receptors (GPCRs), widely manifest in liver resident cells and regulate hepatic activities in pathophysiologically normal conditions, including the metabolism (Jain and Jacobson 2021). Liver diseases are linked to the activation of P2X members. The contribution of P2RX7 in metabolic syndrome has received the most attention, causing hepatocyte apoptosis in mice with a high-fat diet and inducing macrophage-mediated inflammation in sepsis-induced liver injury (Chatterjee et al. 2012; Savio et al. 2017). Studies have reported that the release of extracellular ATP subsequently activated P2RY2, a member of the P2Y purinergic receptor family, stimulated DNA damage responses and hepatocyte proliferation leading to the promotion of hepatocarcinogenesis in mice (Schulien et al. 2020). P2RX1, a member of the P2X purinergic receptor family, also shared similar ligand-gated ion permeability with P2RX7, except for cytokine release requiring a large aperture. Evidence has emerged that P2RX1 contributed to urogenital, immune response, and cardiovascular function (Illes et al. 2021). In addition, the key involvement of P2RX1 on regulating T cell metabolism and neutrophil extracellular traps (NETs) production through mitochondria pathways has been established (Ledderose and Junger 2020).

Since NAPQI principally engages with mitochondrial components, there is hope that the molecules responsible for controlling APAP-induced oxidative stress might be included as a solution to AILI (Qiu et al. 1998). It was postulated that ATP-gated P2RX1 would have been the benchmark of AILI due to the sheer correlation among mitochondrial dysfunction and AILI. After the APAP challenge, mitochondria dysfunction was ameliorated in *P2rx1* deficiency mice which could be verified by in vivo experiments and RNA-seq analysis.

Immune cell infiltration and inflammatory surge are typical pathophysiological roles in APAP overdose (Antoniades et al. 2012). As hepatocyte collapse advances, disrupted cells shed their substances, including fragments of nuclear DNA. This triggers the transcriptional activation of pro-inflammatory cytokines, as well as chemokines, which in turn recruit neutrophils and monocytes to the liver necrosis sites. This immunology response generally contributes to clearing cell debris and promoting liver regeneration (Woolbright and Jaeschke 2015). However, unresolved inflammation also acts as a ﻿detrimental factor in cellular defense against toxicity (Imaeda et al. 2009). ﻿Therefore, targeting inflammation is identified as a therapeutic strategy for APAP overdose. Resolution of bonds to the aggressive component of APAP, which lessened liver damage and prolonged the curative interval eightfold versus the current therapeutic interventions, N-acetyl cysteine, as highlighted by Patel (Patel et al. 2016). ﻿Our findings suggest that P2RX1 represents a valuable position in immunity modulation in AILI, acting as a crucial mediator during this process. AILI recovery has been interconnected to a decrement in inflammatory response brought about by *P2rx1* depletion. This reduction in inflammation manifested itself in a diminishing of neutrophils and macrophage infiltration and a decline in the rollout of inflammatory cytokines. (Liew et al. 2017).

It is worth noting that cGAS is a sensor of aberrant intracellular DNA cyclic catalyzes the synthesis of guanosine monophosphate–adenosine monophosphate (cGAMP), bound by STING dimer (Ishikawa et al. 2009). A signaling cascade triggered by the STING-independent activation of TBK1 would ultimately lead to the P65 engagement (Cai et al. 2020). The activated STING-TBK1-P65 signaling pathway is associated with promoting innate immune response and necrosis of hepatic tissues, participating in multiple types of acute and chronic hepatic injury. Thus, inhibiting the STING signaling pathway in hepatic non-parenchymal cells is a potential therapy for AILI. Mechanically, our study suggests that the protective impact of *P2rx1* depletion on AILI is due, in part, to its ability to keep the STING signaling pathway from being activated when it should be.

In conclusion, our findings provided new evidence of the protective effects of *P2rx1* depletion in AILI and a basis for developing new drugs from natural products since small molecule antagonists of the P2RX1, such as NF449, have been available (Mdawar et al. 2019). Blockade of P2RX1 could be a promising strategy to promote inflammation resolution and mitochondria function through the STING signaling pathway. P2RX1 is closely associated with human liver disorders, however, its pivotal nature and its translational application are yet unclear, calling for extensive exploration.

## Data Availability

The datasets generated for this study are available on request to the corresponding author**.**
